# Acrylamide Occurrence in Iranian Biscuits and Its Potential Risk of Exposure

**DOI:** 10.1002/fsn3.70480

**Published:** 2025-07-08

**Authors:** Saeed Hoseini Majd, Seyed‐Ahmad Shahidi, Nabi Shariatifar, Mohammad Ahmadi, Mahdi Sharifi Soltani

**Affiliations:** ^1^ Department of Food Hygiene, Am.C. Islamic Azad University Amol Iran; ^2^ Department of Food Science and Technology, Am.C. Islamic Azad University Amol Iran; ^3^ Department of Environmental Health Engineering, School of Public Health Tehran University of Medical Sciences Tehran Iran; ^4^ Department of Food Hygiene, Cha.C. Islamic Azad University Chaloos Iran

**Keywords:** acrylamide, cereal‐based products, gas chromatography–mass spectrometry, graphene (G) modified with magnetite, incremental lifetime cancer risk, sol–gel hybrid tetraethoxysilane methyltrimethoxysilane

## Abstract

Acrylamide is a chemical that can form in some foods during high‐temperature cooking processes and is a known carcinogen according to the International Agency for Research on Cancer (IARC). Since biscuit consumption is high amongst Iranians, especially children, and there is a risk of exposure to hazardous compounds such as acrylamide, the purpose of this study is to investigate the amount of acrylamide in some types of biscuits. In this study, graphene (G) modified with magnetite (Fe_3_O_4_) and sol–gel hybrid tetraethoxysilane methyltrimethoxysilane (TEOS‐MTMOS) or MSPE (magnetic solid phase extraction) and GC/MS (gas chromatography–mass spectrometry) method was used to measure acrylamide. The calibration curve, LOD (detection limit), LOQ (quantification limit), RSD (Relative standard deviation), recovery, and linear *r* ^2^ (correlation coefficient) were 0–200 ng/g, 19 μg/kg, 58 μg/kg, 9.64%, 96%, and 0.9982, respectively. The results displayed that the highest level was observed in the biscuits sample with cardamom essential oil (9.01 mg/kg) and the lowest level was observed in the saffron biscuits sample (0.15 mg/kg). According to the results, the mean ± SD of all samples was 1.739 ± 0.830 mg/kg, which being higher than 0.35 mg/kg can be higher than the EU (European Union) standard level. Our results also indicated that biscuits containing essential oil (mean = 4.510 mg/kg) were more contaminated than biscuits containing natural additives (mean = 0.806 mg/kg). Monte Carlo simulation results showed the THQ (Target Hazard Quotient) and ILCR (Incremental Lifetime Cancer Risk) associated with exposure to acrylamide via biscuits for adults were 2.52E‐1 and 2.48E‐4; and for children were 8.75E‐1 and 8.47E‐4, respectively. Uncertain analysis of human health risks by consumption of biscuit samples contaminated with acrylamide showed a serious non‐carcinogenic risk (95th percentile; THQ > 1) for children and a carcinogenic risk (ILCR > 1E‐4) for children and adults. Consequently, there is a significant carcinogenic risk from biscuit consumption.

## Graphical abstract

The acrylamide level in various Iranian biscuits was measured using the MSPE (graphene (G) modified with magnetite (Fe_3_O_4_) and sol–gel hybrid tetraethoxysilane methyltrimethoxysilane (TEOS‐MTMOS))‐GC/MS method.

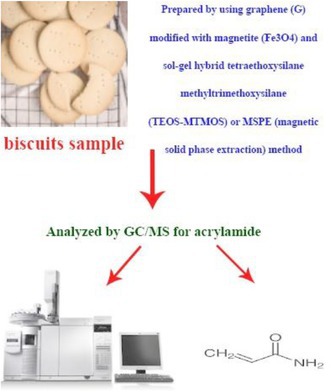

## 1 Introduction

One of the most important sources of human energy is plant food, including cereals, which have been used since ancient times. Cereals have different compounds such as protein, carbohydrates, vitamins, minerals, fat, etc. Cereal products such as biscuits are prepared from wheat flour and other cereals that contain protein, carbohydrates, vitamin E, vitamin B complex, niacin, fiber, carbohydrates, riboflavin, thiamin, iron, and other minerals (Azarashkan, Motamedzadegan, Ghorbani‐HasanSaraei, Rahaiee, et al. 2022). Biscuits are a popular cereal‐based food (wheat flour) and are consumed by a wide range of people due to their different flavors, long shelf life, and relatively low price. The main ingredients of biscuits include flour, sugar, oil, water, etc. (Gazi et al. 2023; Schouten et al. 2022). Some contaminants (Acrylamide) may be produced in some protein and carbohydrate foods such as biscuits during the baking process. Acrylamide is produced in food during baking through a chemical reaction known as the Maillard reaction, which occurs between sugars and amino acids (especially asparagine) when foods are heated at high temperatures (typically above 120°C or 248°F). This process is common in starchy foods like bread and cookies and results in the formation of acrylamide as a byproduct (Biedermann et al. 2010; Matthys et al. 2005; Mojska et al. 2010). Acrylamide is an odorless organic analyte with a molecular weight of 71.08, great solubility in water, a boiling point temperature (136°C at 3.3 kPa/25 mmHg) and 84.5°C ± 0.3°C (Biedermann et al. 2010; Matthys et al. 2005; Mojska et al. 2010).

Acrylamide is a (potentially) toxic analyte with a wide range of adverse effects. One of the most important adverse effects of acrylamide exposure in humans and rodents is neurotoxicity. This combination prevents the differentiation of glioblastoma and neuroblastoma cells in humans (Mousavi Khaneghah et al. 2022; Saleh and El‐Okazy 2007; Svensson et al. 2003). In humans, neurological symptoms of acrylamide include axonal degeneration, skeletal muscle weakness, ataxia, and weight loss. Acrylamide has been labeled as a carcinogenic and genotoxic agent in Iran and other countries. Acrylamide is responsible for the genotoxic effects of a compound called glycidamide, which is a key metabolite of acrylamide (Rufian‐Henares et al. 2007; Saleh and El‐Okazy 2007; Svensson et al. 2003). Various studies have shown that exposure of DNA to varying doses of acrylamide results in high levels of DNA damage. Also, some researchers have predicted a link between long‐term exposure to acrylamide and breast cancer in women. In addition, the effect of acrylamide on the human immune system has been investigated in studies. Acrylamide reduces the number of lymphocytes and leads to abnormalities in the spleen, thymus and lymph nodes (Çebi 2024; Schouten et al. 2022; Seilani et al. 2021; Shahbazi et al. 2022). The European Union has recommended a maximum permissible level of 350 μg/kg for acrylamide in biscuits (European Union 2017).

Techniques for assessing acrylamide in different type of food comprise Liquid chromatography–mass spectrometry (LC–MS), GC–MS, and High‐performance liquid chromatography (HPLC), which amongst them, GC–MS is more used and popular owed to its accuracy, convenience and cheapness (Azarashkan, Motamedzadegan, Ghorbani‐HasanSaraei, Biparva, et al. 2022; Normandin et al. 2013; Svensson et al. 2003). Also, in assessment of human health risk, the use of MCS (Monte Carlo simulation) can solve the inability to attain accurate results owed to the variables' uncertainty. At present, the MCS method has been broadly used in the health risk assessment of human caused by hazardous combinations in foodstuff (Konings et al. 2003; Matthys et al. 2005; Shahbazi et al. 2022). Since biscuits are one of the items in the food basket of Iranian households and their consumption is increasing, and considering the possible dangers of acrylamide formed during baking in this food, it seems necessary to conduct this research to investigate the addition of various additives to reduce acrylamide. Therefore, the purpose of this article was to investigate the effect of several different additives for the first time in Iran in reducing acrylamide levels in various Iranian biscuits using graphene (G) modified with magnetite (Fe_3_O_4_) and sol–gel hybrid tetraethoxysilane methyltrimethoxysilane (TEOS‐MTMOS) or MSPE (magnetic solid phase extraction) and GC/MS (gas chromatography–mass spectrometry) method and its human health risk assessment.

## 2 Materials and Methods

### 2.1 Collection of Biscuit Sample

Forty‐two samples of biscuit (in duplicated and two samples of each biscuit) were obtained from supermarkets in Tehran. Biscuit samples included: biscuit with strawberry EO (essential oil); biscuit with orange EO; biscuit with banana EO; biscuit with forest fruit EO; biscuit with cardamom EO; biscuit without sugar and Non‐GMO (genetically modified) ingredients; biscuit with oat bran; biscuit without sugar; biscuit with sugar and Non‐GMO; whole wheat biscuits without trans fat; biscuit with genetically modified ingredients; biscuit with four nectars and three grains; hazelnut biscuits; vanilla biscuits; cocoa biscuits; milk biscuits; apple and cinnamon biscuits; saffron biscuits; coconut biscuits; cappuccino biscuits; and coffee biscuits. The collected samples were immediately conveyed to the laboratory and were stored in the packaging and under the conditions stated on the packaging until testing.

### 2.2 Reagents and Chemicals

From Sigma Aldrich (St. Louis, MO, USA), standard acrylamide (purity > 99.8%) was acquired. Ethanol (97%), acetone (AR grade), methanol (HPLC grade), and ethyl acetate were ordered from QReC (Selangor, Malaysia). Also, from Merck (Schuchardt, Germany), acetonitrile (ACN) with HPLC grade and n‐hexane were ordered. In methanol, the primary standard solution of acetamide and acrylamide (2000 μg/mL) was prepared. To attain the working solution, the mentioned standard solution was prepared in methanol. Finally, at 4°C, working solutions and stock were reserved (Aghvami et al. 2023).

### 2.3 Preparation of Magnetic Graphene‐Based Hybrid Sol–Gel Material

The Fe_3_O_4_@G‐TEOS‐MTMOS (magnetic material based on graphene modified with magnetite and sol–gel hybrid tetraethoxysilane methyltrimethoxysilane) as adsorbent was prepared as reported in former studies (Nodeh et al. 2017, 2018).

### 2.4 Sample Preparation (Solid–Liquid Extraction and Magnetic Solid Phase Clean‐Up Procedures)

All sample preparation steps were performed according to previous studies (Nodeh et al. 2017, 2018). This step was performed at achieved conditions of optimized (1 g food sample, 2 min extraction time, 5 mL ACN (acetonitrile) as extraction solvent, 70 mg adsorbent and 5 min clean‐up time). One gram of homogenized sample was transferred into a 15 mL centrifuge tube. Next, n‐hexane (5 mL) was added for the defatting process, followed by 2 min of vigorous mixing using a Heidolph vortex shaker (Schwabach, Germany). In order to extract acrylamide from the samples, ACN (5 mL) was added, then vortexed for 2 min, and later centrifuged (for 10 min at 4000 rpm) in a KUBOTA 2420 centrifuge (Tokyo, Japan). Two phases were observed; the n‐hexane phase includes fat (top layer) and the ACN phase (pinkish colored solution), which contained the extracted acrylamide and other soluble analytes, most probably anthocyanins (down layer). The n‐hexane layer was discarded, and the ACN phase was pipetted, then filtered through a 0.45 μm nylon syringe filter (Croydon, Surrey) into a 10 mL glass vial. Next, the prepared adsorbent (70 mg) was added for purposes of clean‐up, followed by shaking the Heidolph vortex for 5 min (clean‐up time). The adsorbent was collected using a magnet (external). Next, 4 mL of the cleaned colorless ACN extract was transferred into another glass vial and evaporated to dryness under a gentle stream of N_2_ gas. The residue was reconstituted with ACN (50 μL) and the reconstituted extract (1 μL) was injected into GC–MS.

### 2.5 Conditions of GC–MS Equipment

All the conditions of the GC device (temperature, time, etc.) were according to the previous study (Aghvami et al. 2023). GC equipment model Agilent 7890A/MS model Agilent 5975c (MSD inert) was applied, and its specifications were as follows: the column (df: 0.25 μm; 95% methyl polyorganosiloxane/5% phenyl siloxane; length × I.D.: 30 m × 0.25 mm) was an Agilent capillary column HP‐5 ms. In this study, the carrier gas was He (helium) with a flow rate of 0.8 mL/min. The injector temperature was 280°C, the injection mode was splitless, and 1 mL was the injection volume. The injector temperature was held at 280°C. The initial temperature of the oven was at 100°C, holding for 1 min, and then the temperature ramp rate was set at 20°C/min to 300°C, holding the temperature for 2 min. The total run time was 21 min. The retention time for the target compound and internal standard was 10.2 and 9.9 min, respectively. The measurement of acrylamide in chosen samples was accomplished based on the mode of Selected Ion monitoring (SIM). Examples of chromatograms are available in Figure S1.

### 2.6 Performance of Method

The calibration curve was drawn from acrylamide standard solution (in methanol) ranging from 0 to 200 (ng/g). The LOD (detection limit) was 19 μg/kg and the LOQ (quantification limit) was 58 μg/kg. The rate of recovery was calculated by spiking three replicates of the real sample of acrylamide ranging from 50 to 500 μg/L. In our study, the recovery rate was 96%. The calibration curve was linear from LOD to 200 μg/kg with the linear *r* ^2^ (correlation coefficient) of 0.9982. The RSD (Relative standard deviation) was evaluated through the investigation of six repetitive analyses of acrylamide, which was 9.64%.

### 2.7 Exposure and Assessment of Health Risk

MCS was applied to analyze human health risks from exposure to acrylamide analyte detected in some types of biscuits. This assessment included the estimation of the estimated daily intake (EDI), along with the assessment of the probability of carcinogenic and non‐carcinogenic risks, based on the methodology introduced by the Environmental Protection Agency (US‐EPA). Likewise, the non‐carcinogenic risk associated with the consumption of acrylamide in manufactured biscuits was evaluated using the Target Hazard Quotient (THQ) and the Incremental Lifetime Cancer Risk (ILCR) based on the following equations:

(1) $$\mathrm{THQ}=\frac{\mathrm{EDI}}{\mathrm{RfD}}$$

(2) $$\mathrm{EDI}=\frac{C\times \mathrm{EDi}\times \mathrm{EFi}\times \mathrm{IR}}{\mathrm{BW}\times \mathrm{AT}}$$

(3) ILCR=CSF×EDI

where EDI is the estimated daily intake; the reference dose of oral analyte for acrylamide was 0.002 mg/kg day (Environmental Protection Agency [EPA] 2022), *C* is the level of acrylamide in the biscuit product (mg/kg dry weight); Edi is the duration (year) of exposure (children = 6 and elders = 30 years) (EPA, 2015); EF is the frequency of exposure (365 days/year) (Kargarghomsheh et al. 2023); AT is the average time (days) (Mehraie et al. 2024); BWi is the average humans body weight (elders = 70 and children = 20 kg) (Karimi et al. 2023); IRi is the rate of ingestion (2 g/day) (Roudbari et al. 2021).

ILCR is the probability of developing cancer by consuming food contaminated with acrylamide. CSF (suggested by the US‐PEA) corresponds to a carcinogenic slope factor of 0.5 (mg/kg/day)^−1^. Moreover, it is necessary to introduce the hazard quotient theory. Theoretically, a HQ index (Hazard Quotient) is a flexible and simple approach, that is, the ratio of potential exposure to a substance to the threshold at which no adverse effects are expected. This allows quick comparisons and is useful for prioritizing further in‐depth risk evaluations (Karami et al. 2023). When THQ < 1, it indicates that the health risk to the human population is acceptable.

### 2.8 Statistical Analysis

The analyzed data (by using the SPSS v. 22.0) were displayed as mean ± SD (standard deviation). In this research, the non‐parametric test of Kruskal–Wallis was used to compare the average amount of acrylamide in the samples. By the software of Crystal Ball (v. 11.1.2.4.600), the MCS investigations were performed (Aghvami et al. 2023).

## 3 Results and Discussion

### 3.1 The Level of Acrylamide in All Biscuit Samples

According to Table 1, which shows the level of acrylamide composition in biscuit samples separately and also shows the statistical analysis (mean, minimum, standard deviation and maximum) of all samples. Based on this table, the highest amount was detected in the biscuit sample with cardamom (essential oil) EO (9.01 ± 0.65 mg/kg) and the lowest level was identified in the saffron biscuits sample (0.15 ± 0.03 mg/kg). According to the mentioned table, the mean ± SD of all samples was 1.739 ± 0.830 mg/kg. Since the standard amount set by the EU for acrylamide compound in wafers and biscuits is 350 μg/kg, except for six types of biscuit samples, the results of our other samples were higher than the established international standards.

**TABLE 1:** The level of acrylamide in biscuit samples (mg/kg).

Types of biscuit	Acrylamide (mean ± SD)	
Biscuit with strawberry EO	2.12 ± 0.19	
Biscuit with orange EO	3.54 ± 0.13	
Biscuit with banana EO	3.21 ± 0.21	
Biscuit with forest fruit EO	4.67 ± 0.32	
Biscuits with cardamom EO	9.01 ± 0.65	
Biscuits without sugar and Non‐GMO (genetically modified) ingredients	0.59 ± 0.03	
Biscuits with oat bran	2.755 ± 0.14	
Biscuits with sugar	0.64 ± 0.05	
Biscuits with sugar and non‐GMO	0.16 ± 0.02	
Whole wheat biscuits without trans fat	0.24 ± 0.04	
Biscuits with genetically modified ingredients	1.53 ± 0.10	
Biscuits with four nectars and three grains	0.48 ± 0.05	
Hazelnut biscuits	0.16 ± 0.03	
Vanilla biscuits	1.29 ± 0.08	
Cocoa biscuits	3.015 ± 0.12	
Milk biscuits	0.96 ± 0.07	
Apple and cinnamon biscuits	0.82 ± 0.04	
Saffron biscuits	0.15 ± 0.03	
Coconut biscuits	0.22 ± 0.02	
Cappuccino biscuits	0.35 ± 0.04	
Coffee biscuits	0.61 ± 0.03	
Statistical analysis of all samples		
	Median	0.820
	Mean	1.739
	Max	9.010
	Min	0.150
	SD	0.830

The higher level of acrylamide compound in biscuit samples may be due to several reasons, such as the use of raw materials with high contamination (flour, sugar, water, milk powder, salt, baking powder, etc.), the use of high heat for baking, and the use of different additives/essential oils (Aghvami et al. 2023; Konings et al. 2003; Matthys et al. 2005).

To reduce the formation of acrylamide in various types of biscuits, several strategies can be implemented. Firstly, using lower baking temperatures and longer baking times can help minimize acrylamide production. Additionally, selecting raw materials with lower asparagine content, such as certain types of flour, can be beneficial. Incorporating ingredients rich in antioxidants, such as certain fruits or vegetables, and natural additives may also help inhibit acrylamide formation. Furthermore, adjusting the pH by adding acidic ingredients like vinegar can reduce acrylamide levels. Lastly, avoiding excessive browning during baking by monitoring color changes can contribute to lower acrylamide content (Aghvami et al. 2023; Konings et al. 2003; Matthys et al. 2005).

Comparison with other studies (Table 2) showed that the amount of this harmful substance (acrylamide) can be different depending on the type of biscuit, baking conditions, and even in different countries.

**TABLE 2:** Comparison with other studies.

Year	Researchers	Food matrix	Results (μg/kg)	References
2003	Konings et al.	Biscuits	< 30–700	Konings et al. (2003)
2005	Matthys et al.	Biscuits	20–1514	Matthys et al. (2005)
2014	Pugajeva et al.	Biscuits	< 10–1060	Pugajeva et al. (2014)
2007	Eerola et al.	Biscuits	< 68–1150	Eerola et al. (2007)
2007	Saleh and El‐Okazy	Biscuits	104	Saleh and El‐Okazy (2007)
2007	Rufian‐Henares et al.	Biscuits	< 30–2085	Rufian‐Henares et al. (2007)
2012	Sirot et al.	Biscuits	697	Sirot et al. 2012
2016	Razia et al.	Biscuits	52.3–507	Razia et al. (2016)
2003	Konings et al.	Crackers	80–420	Konings et al. (2003)
2010	Mojska et al.	Crackers	566–2017	Mojska et al. (2010)
2013	Normandin et al.	Crackers	15–1040	Normandin et al. (2013)
2007	Rufian‐Henares et al.	Crackers	< 30–296	Rufian‐Henares et al. (2007)
2003	Svensson et al.	Wafers and waffles	42	Svensson et al. (2003)
2010	Biedermann et al.	Cookies	400	Biedermann et al. (2010)
2009	El‐Ziney et al.	Cookies	40–350	El‐Ziney et al. (2009)
2007	Saleh and El‐Okazy	Cake and pastries	12	Saleh and El‐Okazy (2007)
2012	Sirot et al.	Cake and pastries	26	Sirot et al. (2012)
2014	Pugajeva et al.	Cake and pastries	20–200	Pugajeva et al. (2014)
2010	Mojska et al.	Cake and pastries	48–672	Mojska et al. (2010)
2023	Aghvami et al.	Cake (cocoa and cinnamon additives)	10.14–212.28	Aghvami et al. (2023)

### 3.2 The Concentration of Acrylamide Compound in Different Groups of Biscuit Samples

Table 3 shows the acrylamide level in three different groups of biscuit samples (biscuit with essential oil, biscuits without additives and biscuit with natural additives). Based on the mentioned table, the highest average level of acrylamide was detected in biscuit samples containing essential oils (4.510 ± 2.440 mg/kg), and the lowest mean concentration of acrylamide was identified in samples containing natural additives (0.806 ± 0.848 mg/kg). The mean amount of acrylamide in a sample without any additives was 0.986 ± 0.935 mg/kg.

**TABLE 3:** The amount of acrylamide in different groups of biscuit samples (mg/kg).

Different groups of samples		Acrylamide
Biscuit with essential oil	Median	3.540
	Mean	4.510
	Max	9.010
	Min	2.120
	SD	2.440
Biscuits without additives	Median	0.615
	Mean	0.986
	Max	2.755
	Min	0.160
	SD	0.935
Biscuit with natural additives	Median	0.545
	Mean	0.806
	Max	3.015
	Min	0.150
	SD	0.846
*p*		0.001

According to the obtained results, it can be stated samples with natural additives have lower acrylamide level (opposite to the addition of essential oils). Also, not adding flavoring is better than adding essential oil. The observed difference in acrylamide levels amongst the biscuit samples can be attributed to the chemical composition of the additives used. Natural additives may contain compounds that inhibit the formation of acrylamide during the baking process, while biscuits without additives lack these protective agents. In contrast, biscuits with flavoring essences may contain precursors that promote acrylamide formation, leading to higher levels. Therefore, the use of natural additives appears to be a more effective strategy for reducing acrylamide content in baked goods compared to the absence of additives or the use of artificial flavoring agents (Aghvami et al. 2023; El‐Ziney et al. 2009; Mousavi Khaneghah et al. 2022; Seilani et al. 2021; Shahbazi et al. 2022).

Hedegaard et al. 2008 showed that the addition of 1% aqueous extract of rosemary with gallic acid (40 mg) and adding rosemary oil or its dried leaves to wheat dough reduced acrylamide contamination by (62, 67, 57) percent, respectively. Several studies have shown that thyme has a lesser effect than rosemary (Hedegaard et al. 2008). Cheng et al. (2009) analyzed the effect of naringenin on chemical models of acrylamide formation and found that despite the weak antioxidant effect of this flavonoid, acrylamide formation was effectively prevented by reaction with precursors. Arribas‐Lorenzo et al. (2009) showed in a cookie system that the amount and composition of the phenolic fraction of olive oil (virgin) significantly affect the formation of acrylamide, especially during long‐term baking. In addition, the use of heat‐sensitive oil (sunflower) increases the amount of acrylamide. They concluded that lipid oxidation products should be mentioned as an important factor in the formation of acrylamide (Arribas‐Lorenzo et al. 2009). Cheng et al. (2010) analyzed extracts of six types of fruits (apple, blueberry, nutmeg, longan, and dragon fruit) in the chemical model of glucose and asparagine and a temperature of 160°C for 30 min to investigate their effect on acrylamide formation. They found that apple extract had an inhibitory effect and, conversely, dragon fruit had an enhancing effect on acrylamide formation, and no significant effect was observed for other fruits (Cheng et al. 2010). Aghvami et al. (2023) analyzed the impact of two additives including cocoa and cinnamon on the formation of acrylamide in samples of cake and expressed that the minimum and maximum average level of acrylamide among samples of cake was associated with the cocoa cake samples (10.14 μg/kg) and the cinnamon cakes (212.28 μg/kg), respectively.

### 3.3 Human Health Risk Assessment

The US‐EPA introduced the MCS (i.e., often employed in stochastic assessment of risk) to estimate the health risk of consuming acrylamide‐contaminated biscuits in children and adults. In Table 4, the EDI index is shown through the consumption of biscuit samples contaminated with acrylamide. The EDI (50th percentile) index (mg/kg body weight per day) of acrylamide‐contaminated biscuits (via oral consumption) was 4.92E‐4 and 1.72E‐3 for adults and children, respectively. The consequence presented the intake of daily was lower than the TDI (Tolerable Daily Intake) for human neurotoxicity (that was 0.04 mg/kg‐day).

**TABLE 4:** The Simulation consequences for frequency and probability of EDI index (mg/kg bw/day) of acrylamide‐contaminated biscuits.

Percentiles	Children (acrylamide)	Adults (acrylamide)
5%	1.15E‐3	3.31E‐4
50%	1.72E‐3	4.92E‐4
75%	2.03E‐3	5.78E‐4
95%	2.60E‐3	7.20E‐4

Several studies have considered daily acrylamide intake, but consistent results have not been obtained due to variations in acrylamide levels, consumption rates, consumption habits, and levels detected in common foods. For instance, in Japan, the mean value of dietary intake (amongst 40–69 year olds) was 0.0068 mg/kg body weight/day (Liu et al. 2019); in France, it was 0.00043 mg/kg body weight/day for adults (European Commission 2011); in Spain, it was 0.00053 mg/kg body weight/day (Delgado‐Andrade et al. 2012); and in Iran, it was 0.0551 mg/kg body weight/day amongst Iranian 11–17 year olds (Mousavi Khaneghah et al. 2022).

**FIGURE 1:**
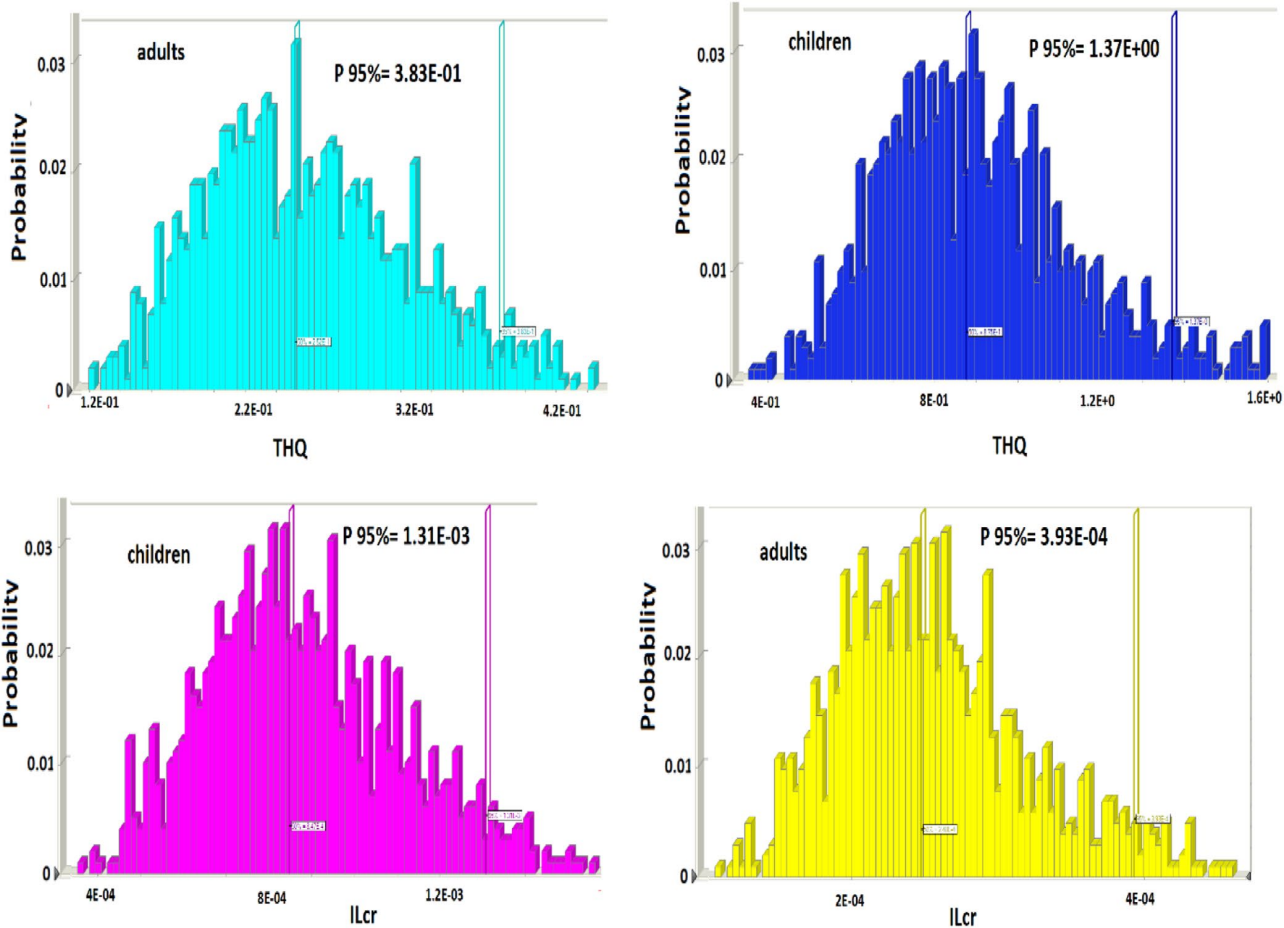
The simulation consequences for frequency and probability of cancer risk (ILCR), and THQ of acrylamide‐contaminated biscuits.

In our research, MCS was used to assess uncertainty in input values, calculate carcinogenic and non‐carcinogenic risk, and also examine probability distributions of exposure simulations. In Figure 1, an assessment of the health risk consequences is displayed. The THQ (50th percentile) index of acrylamide‐contaminated biscuits (via oral consumption) was 2.52E‐1 and 8.75E‐1 for adults and children, respectively. Despite this, the 95th percentile for children was estimated as a possible critical (1.37) endpoint for acrylamide non‐carcinogenic risk. This is supported by the increase (more than niine times) of acrylamide concentration in biscuits with cardamom EO compared to biscuits without additives, which increased the overall average concentration of acrylamide compound in biscuit samples. In our research, Figure 1 shows the frequency distribution of ILCR for children and adults. In the ILCR index, a value less than 1 × 10^−6^ is considered a negligible (insignificant) risk of carcinogenesis for humans and a value greater than 1 × 10^−4^ is considered a significant risk of cancer from harmful pollution. The ILCR (50th percentile) index of acrylamide‐contaminated biscuits (via oral consumption) was 2.48E‐4 and 8.47E‐4 for adults and children, respectively; representing no risks to human health linked to the intake of acrylamide‐contaminated biscuits. Hence, the risk for impacts of carcinogenic human health is significant (> 1 × 10^−4^). Hence, biscuit products (especially with additives) are at considerable risk of carcinogenicity for consumers. Likewise, in Iran, research assessed the human health risk of process‐related acrylamide in breads (Nwoke et al. 2021). Based on the assessment of health risk, the risk indexes of carcinogenic and non‐carcinogenic by consuming food contaminated with acrylamide were dangerous for human health risk (significant) for all consumers in Tehran.

## 4 Conclusion

In our study, the amount of acrylamide in some types of biscuits (biscuits with essential oils, biscuits without additives and biscuits with natural additives) was evaluated using graphene (G) modified with magnetite (Fe_3_O_4_) and the sol–gel hybrid tetraethoxysilane methyltrimethoxysilane (TEOS‐MTMOS) or MSPE/GC–MS technique. The order of the average amount of acrylamide in the form of biscuits with natural additives was < biscuits without additives < biscuits with essential oils and biscuits with essential oils. On the contrary, the highest and lowest amount of acrylamide was related to biscuits with cardamom EO and biscuits with saffron additive, respectively. According to the obtained results, the decrease in the amount of acrylamide was related to the natural additives of biscuits. According to the obtained results, since in some biscuit samples, the amount of acrylamide composition was higher than the European Union standards, the use of raw materials with higher quality and the need for more care during production seems obligatory. One of the limitations of the study is the lack of financial resources to assess the amount of acrylamide in other similar products, for which it is proposed to assess this pollution (acrylamide) in other flour‐based foods such as crackers, wafers, cookies, etc. Uncertain analysis of human health risks by consumption of biscuit samples contaminated with acrylamide showed a serious non‐carcinogenic risk (95th percentile; THQ > 1) for children and a carcinogenic risk (ILCR > 1E‐4) for children and adults. Finally, it is suggested that details of acrylamide compound formation and health risk assessment findings in dietary patterns be included in carefully planned food surveillance data.

## Author Contributions

**Saeed Hoseini Majd:** funding acquisition (equal), software (equal), validation (equal), visualization (equal), writing – original draft (equal). **Seyed‐Ahmad Shahidi:** project administration (equal), resources (equal), software (equal), validation (equal), visualization (equal), writing – review and editing (equal). **Nabi Shariatifar:** conceptualization (equal), supervision (equal), validation (equal), visualization (equal), writing – review and editing (equal). **Mohammad Ahmadi:** conceptualization (equal), data curation (equal), formal analysis (equal), funding acquisition (equal), investigation (equal), methodology (equal), writing – original draft (equal). **Mahdi Sharifi Soltani:** data curation (equal), formal analysis (equal), project administration (equal), resources (equal), software (equal), visualization (equal), writing – original draft (equal).

## Ethics Statement

The authors have nothing to report. This study does not involve any human or animal testing. All authors agree to publish.

## Conflicts of Interest

The authors declare no conflicts of interest.

## Supporting information


**Figure S1.** Samples from GC–MS chromatogram (A = real sample, B = spiked sample).

## Data Availability

The data that support the findings of this study are available from the corresponding author upon reasonable request.
