# Supplementary figures and images for: Acrylamide Occurrence in Iranian Biscuits and Its Potential Risk of Exposure
Source: Food Sci Nutr. 2025 Jul 8;13(7):e70480. doi: 10.1002/fsn3.70480 (PMC12235668; doi:10.1002/fsn3.70480)

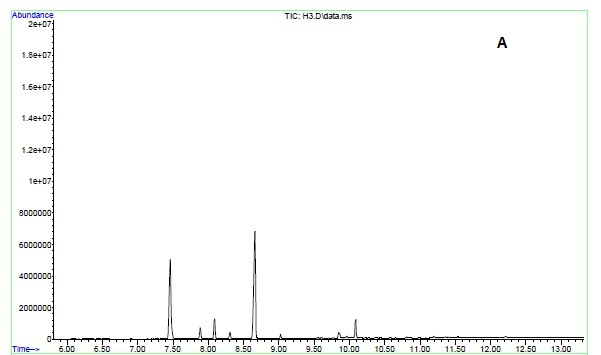


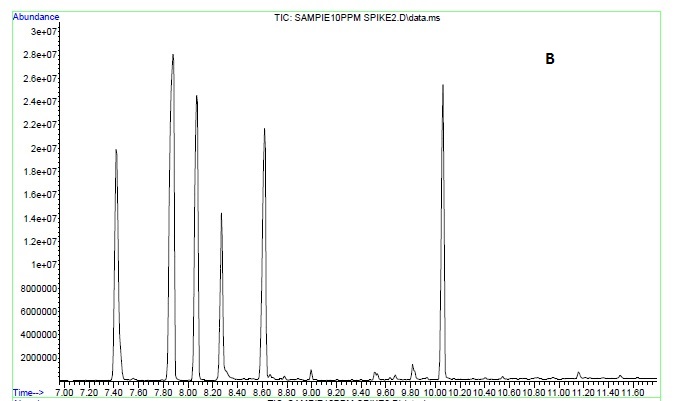


**Figure S1. Samples from GC-MS chromatogram (A=real sample, B=spiked sample).**

Supplement: Supplementary file 1 — Figure S1. Samples from GC–MS chromatogram (A = real sample, B = spiked sample). [file FSN3-13-e70480-s001.docx]
